# Pre-analytical sample handling effects on blood cytokine levels: quality control of a COVID-19 biobank

**DOI:** 10.2217/bmm-2020-0770

**Published:** 2021-07-22

**Authors:** Inge MW Verberk, Esther J Nossent, Hetty J Bontkes, Charlotte E Teunissen

**Affiliations:** 1 Department of Clinical Chemistry, Neurochemistry Laboratory, Amsterdam Neuroscience, Amsterdam UMC, Vrije Universiteit Amsterdam, Amsterdam, The Netherlands; 2 Department of Pulmonary Medicine, Amsterdam Cardiovascular Sciences Research Institute, Amsterdam UMC, Vrije Universiteit Amsterdam, Amsterdam, The Netherlands; 3 Department of Clinical Chemistry, Medical Immunology Laboratory, Amsterdam UMC, Vrije Universiteit Amsterdam, Amsterdam, The Netherlands

**Keywords:** Biobank, interferon, interleukin, pre-analytics, stability, tumor necrosis factor

## Abstract

**Aim:**

We investigated the effect of pre-analytical sample handling variations on coronavirus disease 2019-relevant circulating cytokine levels IFN-γ, IL-10, IL-12p70, IL-17A, IL-6 and TNF-α.

**Materials & methods:**

We collected blood in different collection tubes (ethylenediaminetetraacetic acid, sodium citrate, lithium heparin, serum), and subjected ethylenediaminetetraacetic acid plasma to among others increasing delays in centrifugation or -80°C storage. Six subjects were included in each experimental condition. Cytokine levels were measured in these samples using the Simoa Cytokine 6-plex kit.

**Results:**

Different tube types resulted in different blood cytokine levels. IL-17A and IL-6 levels declined with 3 h centrifugation delay. IFN-γ levels declined with 24 h postcentrifugation storage delay. IL-17A levels declined with 2-week storage delay.

**Conclusion:**

It is recommended to centrifuge tubes quickly following collection, for accurate cytokine measurement.

The novel SARS-CoV-2 virus first appeared in Wuhan, China in December 2019, and has quickly spread over the world. In January 2020, the WHO identified the virus outbreak as a Public Health Emergency of International Concern, in other words, a pandemic. At this time, May 2021, >168,000,000 cases globally were confirmed infected with this coronavirus causing COVID-19, and >3,400,000 COVID-19 deaths were reported [1]. These numbers are still rising, and are expected to rise until sufficiently large global immunity through vaccination is reached.

The severity of COVID-19 disease is very heterogeneous between individuals and ranges from mild symptoms of upper respiratory tract infection in most cases to severe pneumonia and even respiratory failure and death in a subset [2]. Around 15% of hospitalized patients develop COVID-19-related acute respiratory distress syndrome [3,4], a clinical phenomenon marked by development of bilateral pulmonary infiltrates and severe hypoxemia. Most of these patients require invasive mechanical ventilation on an intensive care unit (ICU). Today, effective treatment of the COVID-19 pneumonia or its sequela in the lung is entirely lacking [5]. Factors that govern either progression to severe disease or remission are not well understood, but distinct clinical presentations might be driven by differential immune responses. Most significant predictors of disease severity relate to either activation or suppression of the host immune response and it is hypothesized that an innate immune-mediated ‘cytokine storm’ might be responsible for the toxicity and end-organ damage mediated by COVID-19 infections.

Biobanked COVID-19 patient-samples are key to study and understand the different disease phenotypes, for example, to study as to whether blood cytokine levels can be used to predict a patient’s transition to severe or critical COVID-19 disease, as well as a patient’s response to therapeutics. Normally, in *de novo* biobanks, the process of sample collection, processing and storage is highly controlled, meaning that all samples are pre-analytically handled in a comparable way [6]. Under the current pressing situation pertaining to patient as well as care-personnel burden, the requirements for high-quality body fluid biobanking are compromised. At several sites, including Amsterdam UMC, The Netherlands, biobanks have been set up to store blood (plasma, serum) samples that are left over after performance of routine diagnostic analyses. This method of biobanking is called further use biobanking, and has as a great advantage to quickly store large numbers of COVID-19 patient samples, in order to promptly answer urgent research questions. However, the effect of variation in pre-analytical processing procedures on biomarkers in samples of such a further use biobank is unknown. We therefore investigated the effect of variations in pre-analytical sample handling on blood-based COVID-19-relevant cytokine levels, within a high-sensitivity immunoassay multiplex panel that has the potential to detect changes in cytokine levels in early stages of the disease course.

## Materials & methods

### Volunteer recruitment & sample collection

Individuals aged >18 years that presented at the Clinical Chemistry department of the Amsterdam UMC, VUmc hospital in the period of 14 October 2019 until 20 January 2020 for any diagnostic blood draw, were asked for their willingness to donate additional blood for research purposes. Following written informed consent to use biomaterials for research purposes, a minimum of two and a maximum of five additional whole blood tubes were collected for our study via venipuncture. These tubes were treated according to one of eight experimental protocols that are described in more detail below. Six independent sample sets for each of the eight experimental protocols were generated and stored at -80°C until analyses. The study was approved by the medical ethical committee of the VU University medical center and was in accordance with the Helsinki Declaration of 1975.

### Sample handling protocols for the generation of pre-analytical sample sets

A reference condition was included in each experimental sample set, which was a blood sample collected into a 6-ml plastic whole blood tube spray-coated with 10 mg K_2_ ethylenediaminetetraacetic acid (EDTA; ref.: 367864, BD Vacutainer, USA), which was left at room temperature (RT) for 30 min, centrifuged at 1800× *g* at RT for 10 min, immediately divided into 250 μl aliquots in 0.5-ml polypropylene storage tubes (ref.: 723730003, Sarstedt, Germany) and stored at -80°C. For the experimental conditions, we systematically varied one of the pre-analytical sample handling steps, while keeping all other sample handling steps comparable to the reference condition. The eight experimental protocols addressed different variables:

1. Blood collection tube type: blood was collected in EDTA tubes (reference), or in 3.2% sodium citrate tubes (ref.: 363048, BD Vacutainer), lithium heparin tubes (ref.: 368886, BD Vacutainer) or gel-separator serum tubes (ref.: 367955, BD Vacutainer);
2. Precentrifugation delay at RT: a delay was applied between collection of EDTA plasma and centrifugation, of 30 min (reference), 1, 3 or 24 h. All tubes were kept at RT during this experiment;
3. Precentrifugation delay at 2–8°C: same as under precentrifugation delay at RT, but here all EDTA blood tubes (including the reference sample) were kept in the fridge (2–8°C) during this experiment;
4. Centrifugation temperature: centrifugation was performed at RT (reference) or at 4°C;
5. Storage tube filling: samples were aliquoted in portions of 250 (reference), 500 or 1000 μl volumes, all in 1.5-ml polypropylene tubes (ref.: 72.703, Sarstedt, Germany);
6. Postcentrifugation delay at RT and 2–8°C: samples were immediately stored (reference) or left at RT or in the fridge (2–8°C) for 4 or for 24 h after centrifugation and aliquoting;
7. Two-week storage stability at low temperatures: samples were stored immediately at -80°C (reference) or stored in the fridge (2–8°C) or at -20°C for 2 weeks;
8. Repeated freeze–thawing: samples underwent no (reference), one, two or four additional freeze–thaw cycles after storage at -80°C. The freeze–thawing protocol was as follows: samples were stored for at least 24 h at -80°C, next thawed at room temperature, mixed by inverting and stored again at -80°C pending analysis, or a subsequent freeze–thaw cycle.

### Measurement of cytokines

Samples were measured with the commercially available Simoa^®^ Human Cytokine 6-Plex Panel 1 Advantage Kit (Quanterix, KY, USA), on board of the Simoa HD-X analyzer. This 6-plex assay simultaneously measures cytokine levels IFN-γ, IL-10, IL-12p70, IL-17A, IL-6 and TNF-α. Samples were measured according to manufacturer’s instructions and blinded for the conditions.

### Data analysis

Data were explored using SPSS for Windows version 22 (IBM), and plots were constructed using R version 3.4.2. Cytokine levels were normalized against their reference condition. We did not perform formal statistical analyses due to the limited sample size, and non-normal distribution of the data. Instead, group medians per experimental protocol and condition were calculated. A median of an experimental condition that differed ≥15% from the reference condition was considered a significant difference.

## Results

The absolute levels (pg/ml) of the cytokines IFN-γ, IL-10, IL-12p70, IL-17A, IL-6 and TNF-α measured in the samples are presented in Table 1. Levels of all six cytokines were above the lower limit of detection for the reference samples. Considering all samples of all experimental conditions, IFN-γ level was below lower limit of detection in one sample (out of 162 measurements). Average % coefficient of variation (%CV) of the duplicate measurements of all samples was highest for IFN-γ, with 20%CV. Also, the highest number of samples with a variation in duplicate measurements >15%CV was observed for IFN-γ (*i.e.*, 32%; Table 1).

**Table 1.** Absolute cytokine levels and analytical variation in the samples.

Cytokine	LLOD (pg/ml)	Reference samples only (n = 46)^†^			All samples (n = 162)^†^		
		Median (IQR) (pg/ml)	Average duplicate %CV	%n with %CV >15	Median (IQR) (pg/ml)	Average duplicate %CV	%n with %CV >15
IFN-γ	0.008	0.15 (0.09–0.30)	22%	44%	0.16 (0.11–0.30)	20%	32%
IL-10	0.003	0.81 (0.59–1.15)	8%	13%	0.81 (0.61–1.16)	8%	10%
IL-12p17	0.002	0.13 (0.09–0.20)	12%	27%	0.12 (0.09–0.20)	13%	24%
IL-17A	0.003	0.10 (0.06–0.19)	16%	33%	0.09 (0.06–0.19)	16%	26%
IL-6	0.017	1.45 (0.81–2.28)	10%	10%	1.45 (0.81–2.28)	9%	11%
TNF-α	0.018	2.04 (1.52–3.00)	9%	15%	2.14 (1.70–2.75)	9%	13%

Median (interquartile range: 25th–75th percentile) cytokine levels (in pg/ml) are presented for the reference samples only, and for all analyzed samples. Average % coefficient of variation of the duplicate sample measurements is reported. LLOD was reported by the manufacturer.

† n = 15 of the reference samples and n = 61 of all samples had a monoplo result only due to technical reasons, therefore, %CV columns are based on a smaller n.

CV: Coefficient of variation; IQR: Interquartile range; LLOD: Lower limit of detection.

### Effect of blood collection tube type

Comparing the analytical precision for each sample type (Table 2), we observed that the average %CV of the duplicate sample measurements was lowest in sodium citrate samples for IL-12p70 (9%CV), lowest in lithium heparin samples for IFN-γ (6%CV), IL-10 (3%CV) and TNF-α (3%CV), and lowest in serum samples for IL-17A (7%CV) and IL-6 (4%CV). For none of the cytokines, the %CV of the duplicate sample measurements was lowest in EDTA. The average %CV of the duplicate measurements for all cytokines ranged from 6 to 22%CV in EDTA samples, from 6 to 36%CV in sodium citrate samples, from 3 to 23%CV in lithium heparin samples and from 4 to 15% in serum samples.

**Table 2.** Measurement precision for each sample type.

Blood collection tube	Average duplicate %CV					
	IFN-γ (%)	IL-10 (%)	IL-12p70 (%)	IL-17A (%)	IL-6 (%)	TNF-α (%)
EDTA	22	7	17	14	18	6
Sodium citrate	36	13	9	18	6	11
Lithium heparin	6	3	17	23	18	3
Serum	15	5	13	7	4	10

Average % coefficient of variation of the duplicate sample measurements is reported for each cytokine for each sample type.

CV: Coefficient of variation; EDTA: Ethylenediaminetetraacetic acid.

**Figure 1.**
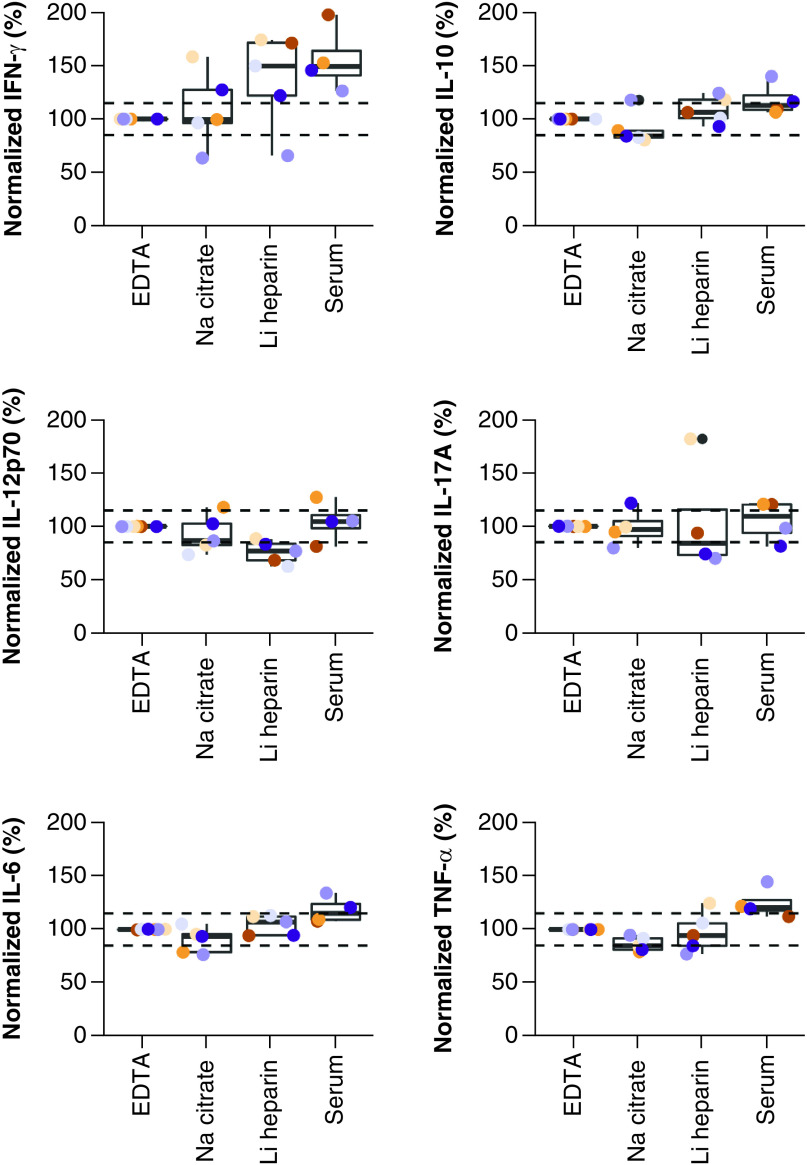
Cytokine levels in samples collected in different whole blood collection tubes. Blood samples were collected in different tubes. Levels were normalized against the reference sample, in other words, levels measured in EDTA plasma (n = 6). Data points are color coded per subject. Horizontal reference lines are fixed at 85 and 115%. EDTA: Ethylenediaminetetraacetic acid; Li: Lithium; Na: Sodium.

Overall, we observed that levels for four out of the six cytokines collected in either sodium citrate or lithium heparin samples were comparable to EDTA samples (the reference conditions) and three out of the six cytokine levels in serum samples were comparable to EDTA samples (Figure 1). Between the sample types, the largest differences between the cytokine levels are noted between sodium citrate and serum samples, notably for IL-10, IL-6 and TNF-α (Figure 1).

More in detail, compared with levels in EDTA samples, median IFN-γ levels were comparable in sodium citrate samples, but were higher in lithium heparin (+50%) and in serum samples (+49%). Median IL-10 levels were lower in sodium citrate samples (-16%) compared with levels in EDTA samples, while levels in lithium heparin samples and in serum samples were comparable to those measured in EDTA. Compared with levels in EDTA samples, median IL-12p70 levels were comparable in sodium citrate samples, were lower in lithium heparin samples (-23%), and were comparable in serum samples. Median IL-17A levels were comparable to levels in EDTA samples in all investigated sample types. Median IL-6 levels were comparable to levels in EDTA samples in sodium citrate samples and lithium heparin samples, but were higher in serum samples (+15%). Median TNF-α levels were lower in sodium citrate samples (-15%), comparable in lithium heparin samples, and higher in serum samples (+21%) compared with levels in EDTA samples.

### Effect of precentrifugation delay at RT or in the fridge

**Figure 2.**
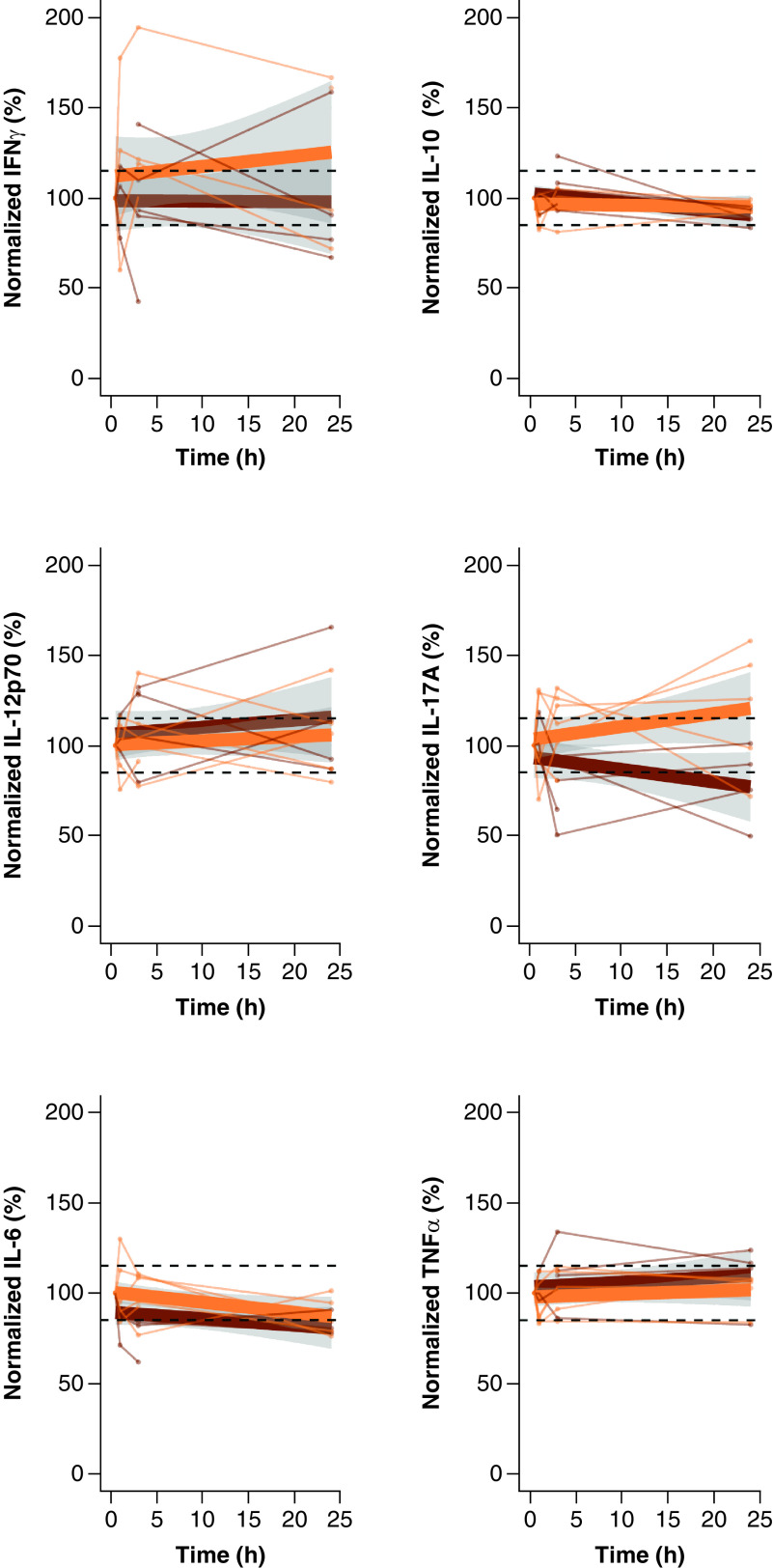
Cytokine levels in EDTA plasma samples with increasing precentrifugation standing times at RT or 2–8°C. EDTA samples underwent delayed centrifugation (1, 3 or 24 h delay), while kept at RT or at 2–8°C. Levels were normalized against the reference sample (30 min standing, at RT or 2–8°C). Lines are plotted per individual (n = 5 for RT; n = 6 for 2–8°C) color coded for standing temperature (RT = dark orange, 2–8°C = light orange), with superimposed average linear slopes (fat lines) and 95% CI (in gray shade) for each standing temperature. Horizontal reference lines are fixed at 85 and 115%. EDTA: Ethylenediaminetetraacetic acid; RT: Room temperature.

Predicted linear slopes of the effect of delayed centrifugation (0.5 h vs 1, 3 or 24 h; at RT or at 2–8°C) of whole EDTA blood tubes are presented in Figure 2.

Upon exploration of the median cytokine levels at each standing time, we observed that IFN-γ levels were the same up to time point 3 h and were decreased at standing time 24 h (-16%) when tubes were kept at RT pending centrifugation. At 2–8°C median IFN-γ level was increased at standing time 1 h (+52%) and remained stably high up to standing time 24 h (at 3 h: +58%; at 24 h: +61%).

Median IL-10 levels were the same up to 24 h, both when tubes were kept at RT or at 2–8°C pending centrifugation. Median IL-12p70 level appeared transiently increased at 3 h (+28%) when tubes were kept at RT pending centrifugation, but levels were comparable to the reference again at 24 h RT. At 2–8°C, levels were the same up to 24 h. Median IL-17A levels were the same after 1 h, but levels were decreased at 3 h (-19%) and 24 h (-18%) when tubes were kept at RT pending centrifugation. By contrast, when tubes were kept at 2-8°C, median IL-17A level was increased at 3 h (+17%) and 24 h (+26%) standing times. Median IL-6 levels were decreased at 3 h (-16%) and 24 h (-20%) standing times when tubes were kept at RT pending centrifugation. When tubes were kept at 2–8°C pending centrifugation, median IL-6 level was decreased at 24 h standing time (-16%). Median TNF-α levels was higher at standing time 24 h when tubes were kept at RT pending centrifugation (+15%), while at 2–8°C levels were the same up to 24 h.

### Effect of centrifugation temperature

Centrifugation of whole-blood EDTA tubes at 4°C was compared with centrifugation at RT. Increased levels of IL-17A (+22%) and TNF-α (+27%) were measured in the 4°C condition compared with the RT condition, while cytokine levels IFN-γ, IL-10, IL-12p70 and IL-6 were comparable (Supplementary Figure 1).

### Effect of tube filling

When cytokine levels of samples stored in aliquot volumes of 500 or 1000 μl were compared with samples stored in aliquot volumes of 250 μl, no differences were observed for any of the cytokines (Supplementary Figure 2).

### Effect of postcentrifugation, prestorage delay at RT & in the fridge

Delay in storage for either 4 h or for 24 h postcentrifugation and aliquoting, either at RT or at 2–8°C was compared with immediate storage at -80°C. IFN-γ levels were affected, while IL-10, IL-12p70, IL-17A and IL-6 remained the same (Supplementary Figure 3). In detail: median IFN-γ level was decreased in the samples that were kept at 2–8°C for 24 h (-23%; note: large variation in this condition, with two samples increased, three samples decreased and one sample with comparable level to their reference samples) only, and thus not at 4 h when kept at 2–8°C, nor at any of these time points at RT.

### Effect of 2-week intermediate storage at low temperatures

**Figure 3.**
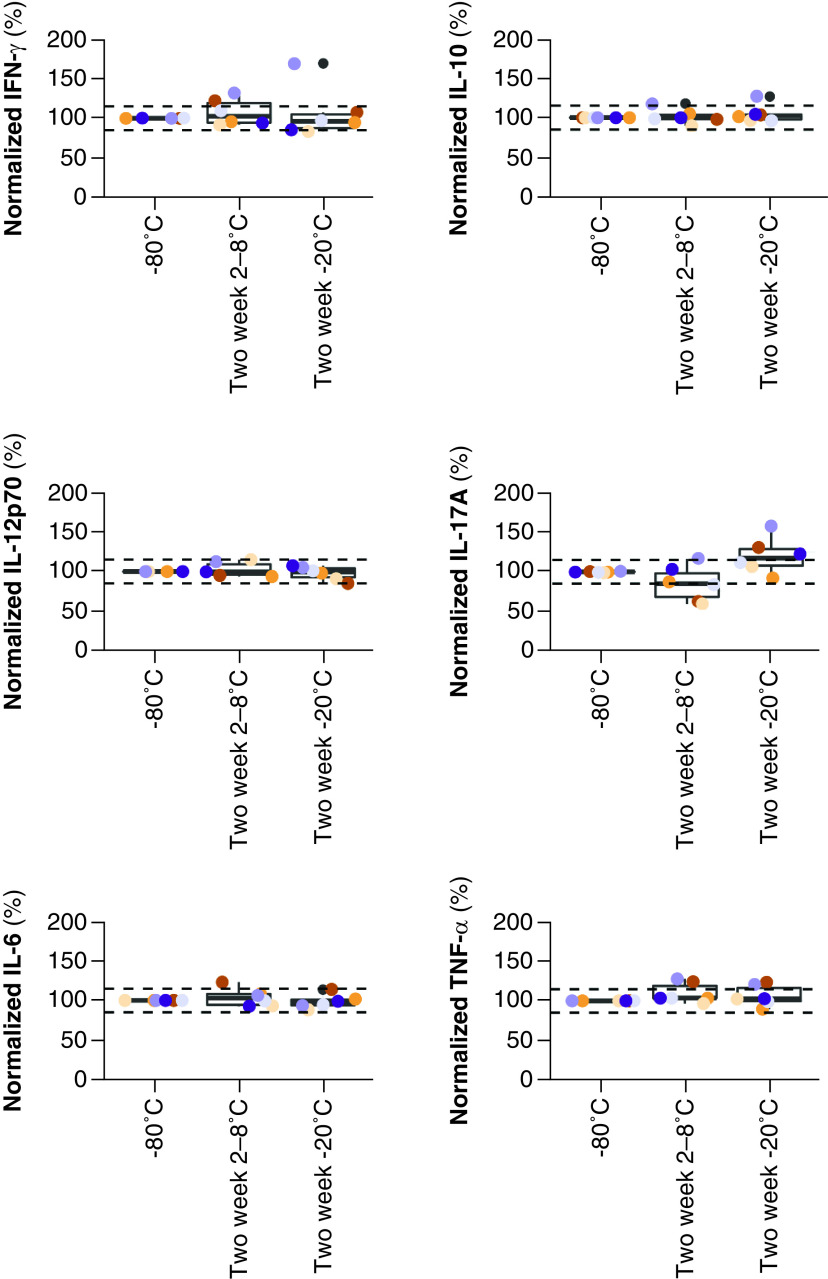
Cytokine levels in EDTA plasma samples upon 2-week intermediate storage at low temperatures. EDTA samples were stored at 2–8°C or -20°C for 2 weeks postcentrifugation and aliquoting. Levels were normalized against the levels measured in the samples that were stored immediately at -80°C. Data points are color coded for subject (n = 6). Horizontal reference lines are fixed at 85 and 115%. EDTA: Ethylenediaminetetraacetic acid.

When aliquots were stored at 2–8°C or at -20°C for 2-weeks (Figure 3), instead of immediate storage at -80°C, only IL-17A cytokine levels were marginally affected, while IFN-γ, IL-10, IL-12p70, IL-6 and TNF-α levels remained stable. Median IL-17A level was decreased in the samples that were kept at 2–8°C for 2 weeks (-15%) pending -80°C storage, while median IL-17A was increased in the samples that were kept at -20°C for 2 weeks (+17%).

### Effect of repeated freeze–thawing

Up to four cycles of additional freeze–thawing prior to thawing for cytokine measurements did not affect any of the cytokine levels IFN-γ, IL-10, IL-12p70, IL-17A and IL-6 and TNF-α (Supplementary Figure 4).

## Discussion

**Figure 4.**
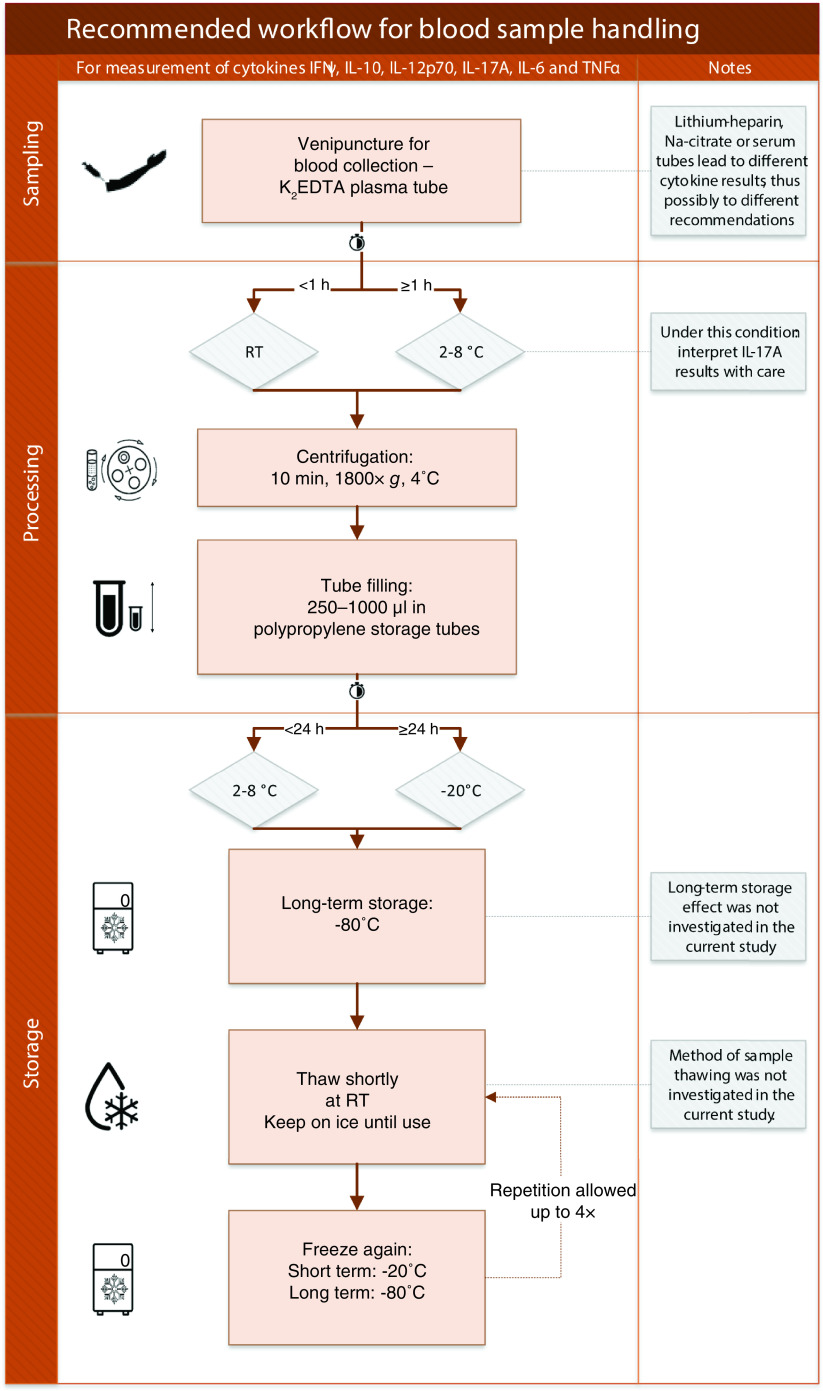
Recommended standardized operating procedure for pre-analytical sample handling for reliable cytokine measurement in blood. Na: Sodium; RT: Room temperature; SOP: Standardized operating procedure.

In this study, we investigated the effect of pre-analytical sample handling variations on levels of COVID-19-relevant cytokines IFN-γ, IL-10, IL-12p70, IL-17A, IL-6 and TNF-α. We observed that in different sample types, the absolute levels of various cytokines were different. Further, we observed that most cytokines were resistant to extended processing times. A negative effect of a delay before centrifugation when standing at RT was observed for three out of the six investigated cytokines (*i.e.*, IL-17A, IL-6, TNF-α). However, when keeping tubes cold pending centrifugation (in the fridge), this negative effect on IL-6 was postponed and the negative effect on TNF-α was prevented. Additionally, only IL-17A from all investigated cytokines was affected by 2-week storage in the fridge (decreased levels) or at -20°C (increased levels) postcentrifugation. Repeated freezing–thawing and differences in aliquot sizes did not affect any of the investigated cytokines. In summary, based on the findings in this study we would recommend to use only one sample type within one study that addresses a blood cytokine level-related research question. Based on our sample type comparison, serum is most likely optimal, with somewhat higher levels for most cytokines and acceptable measurement precision for all cytokines. When sample processing within half an hour is not possible, we would recommend keeping the tubes in the fridge pending centrifugation. When immediate storage at -80°C following centrifugation is not possible, aliquots can be kept either at RT or in the fridge for a short duration (up to 24 h), and in the fridge or at -20°C for longer durations, although, in this case, IL-17A results should be interpreted with care. Our recommended pre-analytical sample handling procedure for accurate cytokine measurement is presented in Figure 4.

Only few previous studies formally investigated cytokine levels under pre-analytical sample handling [7–10]. Three of these studies focused on delayed whole-blood centrifugation [7–9]. One study compared up to 24 h delayed EDTA plasma centrifugation while tubes were kept either at RT or in the fridge, and reported that IFN-γ, IL-6 and TNF-α remained stable both at RT and in the fridge [9]. In contrast, we observed decreasing IL-6 levels both in the RT and fridge condition. Another study investigated EDTA plasma centrifugation delayed up to 3 days while keeping tubes at RT, and showed, contradictory to our findings, that IFN-γ decreased with 75%/day whereas IL-6 remained stable [8], but comparable to our findings that IL-10, IL-12p70 and TNF-α levels remained stable (stability IL-17A not reported) [8]. A last, larger study (n = 88) investigated IL-17A precentrifugation stability at RT, and reported that IL-17A remained stable after a processing delay of 48 h [7]. By contrast, we found decreased IL-17A levels at time points 3 and 24 h precentrifugation. One study investigated the effect of delayed storage postcentrifugation, and observed that cytokine levels IL-6, TNF-α and IFN-γ remained stable up to 1 month storage in the fridge [10], in line with our observation at 2-week postcentrifugation storage in the fridge. Taken together, these conflicting findings indicate that studying pre-analytical sample handling effects on cytokine levels is not a straightforward process. It is worthy of note that all studies used different experimental, analytical as well as statistical methods. Sample size was smaller in our study compared with the previous studies (*i.e.*, n = 10 [8], n = 16 [9] and n = 88 [7]), which might explain some of the conflicting findings between studies. Two of the previous studies specified that healthy volunteers were included [8,9]. Based on the low cytokine levels measured in our samples obtained from volunteers from an outpatient clinic, we judge to have included healthy individuals as well. Still, there were large differences in the absolute concentrations between the previous studies [8,9], as well as between the previous studies and our current study. Particularly, reported levels of IFN-γ (*i.e.*, averages of 63 [9] or 7 pg/ml, compared with our median 0.16 pg/ml) and TNF-α (*i.e.*, averages of 16 [9] or 6 pg/ml [8], compared with our median 2 pg/ml) were very different. Differences in laboratory methods between studies might explain some of the conflicting findings as well. We applied the novel high-sensitivity cytokine panel developed by Quanterix, while the other studies used other analytical approaches (*i.e.*, Luminex xMAP multiplex technology [7,8] or ELISA [9]). We assumed that highest assay sensitivity is required to accurately investigate small, but relevant differences between sample handling conditions. Even though we applied a high-sensitivity assay, accurately measuring the ultra-low levels in cytokines in blood remained challenging. For example, for IFN-γ measurements, 44% of the measurements had an intra-assay variation of %CV >15. One example where this analytical variation might have influenced our results is in our observation of decreased IFN-γ level at 24 h postcentrifugation storage delay in samples that were kept in fridge, but counterintuitively, not in the samples that were kept at RT for 24 h, or in the samples that were kept in the fridge for 2 weeks. From the graphs it can clearly be observed that between-subject variation was large in this experimental condition, therewith impacting the median level. Repetition of the experiments with ambiguous findings in larger sample sizes might be recommended.

In this study, we investigated pre-analytical sample handling variables as they might occur in further use biobanking procedures. Our study results suggested that precentrifugation delays have the highest impact on blood cytokine levels. In further use biobanks, sample processing times are often increased. Delays are due to the routine diagnostic sample measurements that have to be performed first, before samples are released for biobank storage. In our center, whole blood EDTA plasma tubes are collected from COVID-19 patients for performance of whole-blood diagnostic analyses such as biomarkers of fibroproliferation or biomarkers of host-immune responses including chemokines and cytokines involved in the activation of neutrophils, lymphocytes, monocytes and the complement system. As a result, the biobanked EDTA plasma samples might have suffered from centrifugation delays of up to 4 h while kept at RT. Under this condition (*i.e.*, as extrapolated from our 3 h, RT precentrifugation delay condition), median IL-12p70 level was >16% increased, IL-17A was >27% decreased and IL-6 was >17% decreased. IFN-γ, IL-10 and TNF-α levels would probably not be affected under this condition. It is important to note that these % deviations were calculated from samples with low cytokine levels. It was reported that plasma cytokines IFN-γ, IL-10, IL-12p70, IL17A, IL-6 and TNF-α levels are increased in COVID-19 patients admitted to the ICU when compared with healthy controls [11]. In patients with severe COVID-19, serum IL-6 levels were even reported to be increased tenfold compared with nonsevere COVID-19 patients [12]. We performed a preliminary analysis ourselves in EDTA plasma samples from ICU patients with COVID-19 using the Simoa 6-plex cytokine assay (data not shown), which confirmed that median IL-6 level is >tenfold higher in ICU patients as compared with the samples used in the current study, while the other cytokines were between twofold and fourfold higher in the ICU samples compared with the samples of the current study. As a cutoff, we chose a deviation of 15% of the median as a relevant deviation. Since the increase in cytokine levels between patients with severe and nonsevere COVID-19 is more than 15%, likely the observed delayed processing effects will not impact the scientific conclusions on COVID-19-related research questions in a further use biobank. Of note, in the current study, we could not investigate if pre-analytical effects are independent of samples’ starting concentrations, in other words, if a comparable, percent-wise effect will be observed among samples with high compared with samples with low concentrations. This remains a subject of investigation for follow-up studies.

Among the strengths of the current study is that we applied a novel high-sensitivity panel test on the ultrasensitive Simoa HD-X technology. Cytokine levels in blood are low, and with this high-sensitive, multiplex technique we were able to measure quickly and cost-effectively a broad panel of cytokines in all samples. Furthermore, our findings are not only relevant for COVID-19, but also for other immune-related diseases. A limitation is that intra-assay precision of the cytokines measurements exceeded 15% CV for several measurements, particularly in IFN-γ measurements, but also to a somewhat lesser extent in IL-17A and IL-12p70 measurements. Moreover, we investigated pre-analytical effects on cytokine levels in a relatively small sample size (n = 6 per experiment), without COVID-19 patients. At last, our collection tube type comparison study should be extended. Serum is obtained after clotting, while plasma is collected in tubes with an anticoagulant that prevents clotting. Serum generally has a lower total protein content as compared with plasma, which might make detection of low-abundant proteins of interest less challenging, but the clotting process might as well affect the proteins of interest [13]. On the other hand, anti-coagulants might interfere in the assays that measure the proteins of interest [13]. Our tube type comparison data suggests that serum might be the preferred matrix for cytokines measurement, as compared with the anticoagulant types EDTA, lithium heparin and sodium citrate. The effect of pre-analytical sample handling variation on cytokine levels in serum remains to be investigated.

## Conclusion

To conclude, the further use biobank method seems suitable for investigating COVID-19 cytokine-related research questions, but variation on IL-17A, IL-6 and IL-12p70 levels might have been introduced by the applied delay in processing. Extending our current study with other markers could further validate the quality of the further use biobank we have set up at Amsterdam UMC for COVID-19, and will also unravel if the processing protocols such as under further use biobanks can be applied for diseases other than COVID-19.

**Summary points**

- Understanding pre-analytical sample handling effects on blood-based cytokine levels helps interpreting cytokine levels in health and disease.
- Cytokines IFN-γ, IL-10, IL-12p70, IL-17A, IL-6 and TNF-α can be accurately measured in blood samples using the high-sensitivity Simoa platform.
- Between different sample types, the largest differences between absolute cytokine levels were noted between sodium citrate and serum samples, notably for IL-10, IL-6 and TNF-α.
- Most cytokines were relatively resistant to extended processing times. A negative effect of a delay until centrifugation when standing at room temperature was observed already at 3 h for cytokines IL-17A and IL-6. By keeping the tubes cold pending centrifugation (in the fridge), the negative effect on IL-6 was postponed.
- Only IL-17A from all investigated cytokines was affected by 2-week storage in the fridge (decreased levels) or at -20°C (increased levels) postcentrifugation.
- Repeated freezing–thawing and differences in aliquot sizes did not affect any of the investigated cytokines.
- Overall, we concluded that most cytokines were relatively stable to pre-analytical sample handling variations.
