# Supplementary material for: Pre-analytical sample handling effects on blood cytokine levels: quality control of a COVID-19 biobank
Source: Biomark Med. 2021 Jul 22;15(12):987–97. doi: 10.2217/bmm-2020-0770 (PMC8359910; doi:10.2217/bmm-2020-0770)
Supplement: Supplementary file 1 [file bmm-15-987-s1.docx]

**SUPPLEMENTARY FIGURES**

***
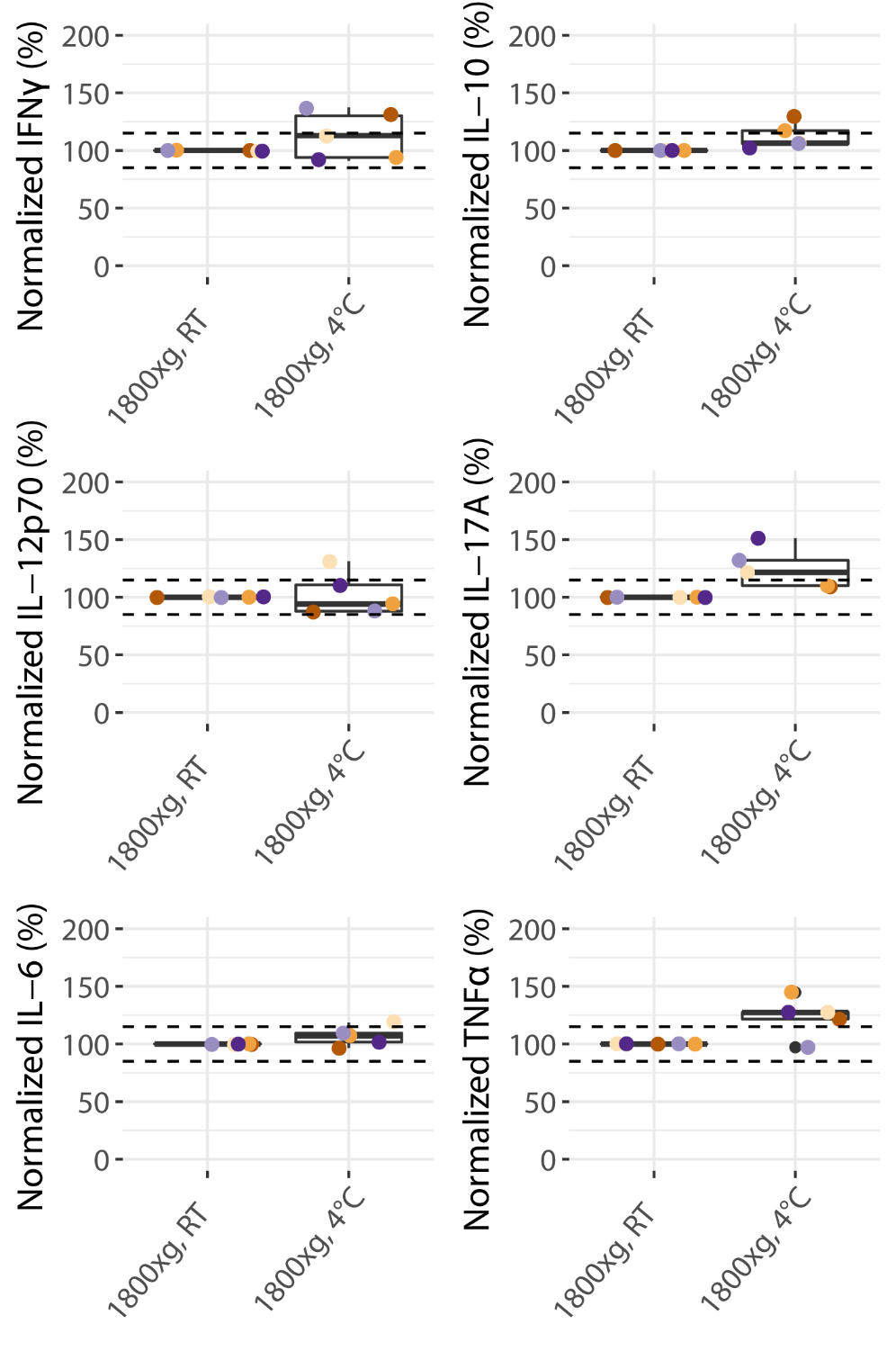
***

**Supplementary figure 1. Cytokine levels in EDTA plasma samples centrifuged at RT or at 4°C.**

*EDTA samples were centrifuged at RT or at 4°C. Levels were normalized against the reference samples (centrifugation at RT). Data points are color coded for subject (n=5). Horizontal reference lines are fixed at 85% and 115%. IFN=interferon, IL=interleukin, TNF=tumor necrosis factor, RT=room temperature.*

**
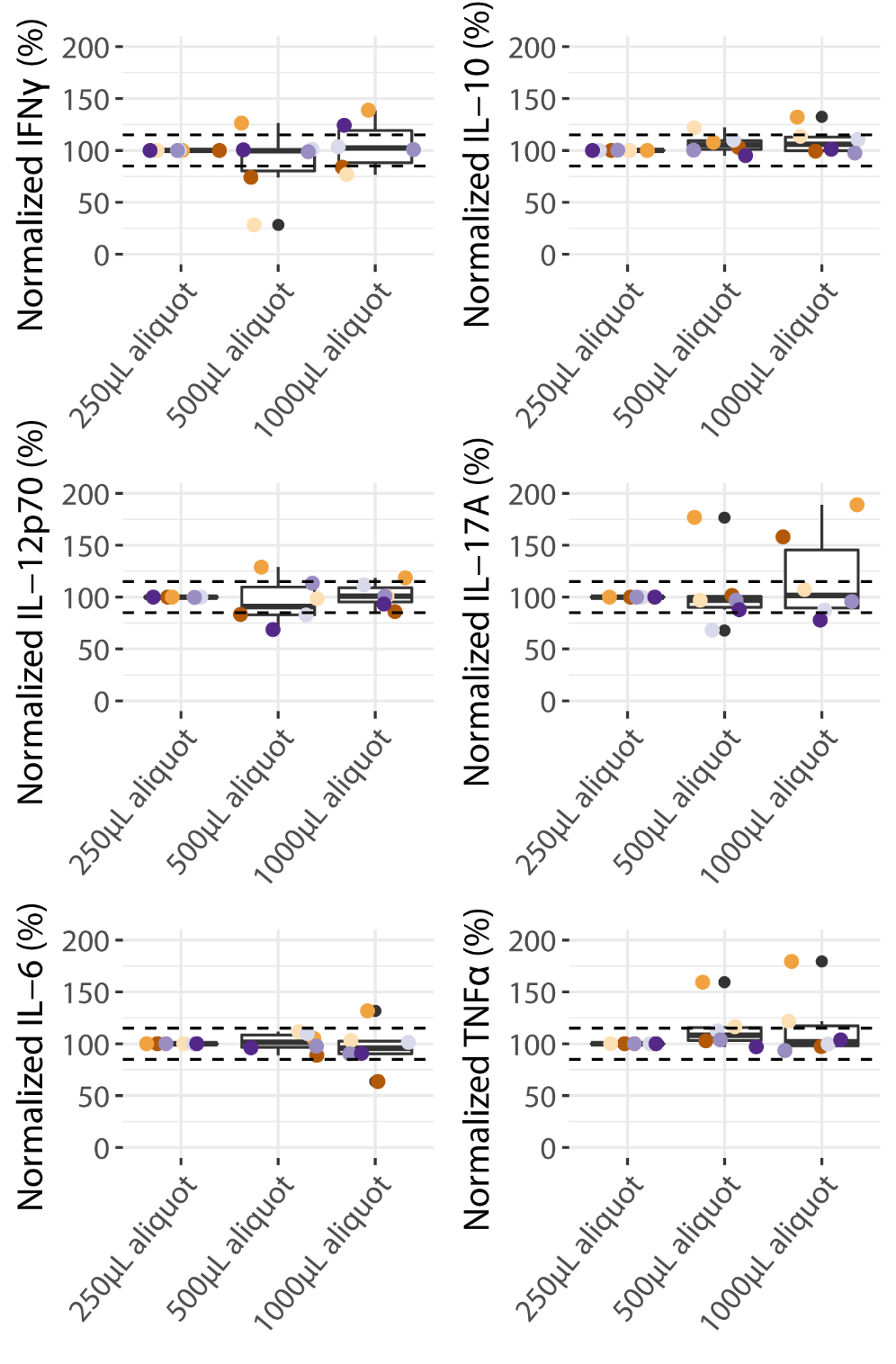
**

**Supplementary figure 2. Cytokine levels in EDTA plasma samples that were stored in aliquot sizes of 250µL, 500µL or 1000µL.**

*EDTA samples were stored in aliquot sizes of 250µL, 500µL or 1000µL. Levels were normalized against the levels measured in the 250µL aliquots. Data points are color coded for subject (n=6). Horizontal reference lines are fixed at 85% and 115%. IFN=interferon, IL=interleukin, TNF=tumor necrosis factor.*

***
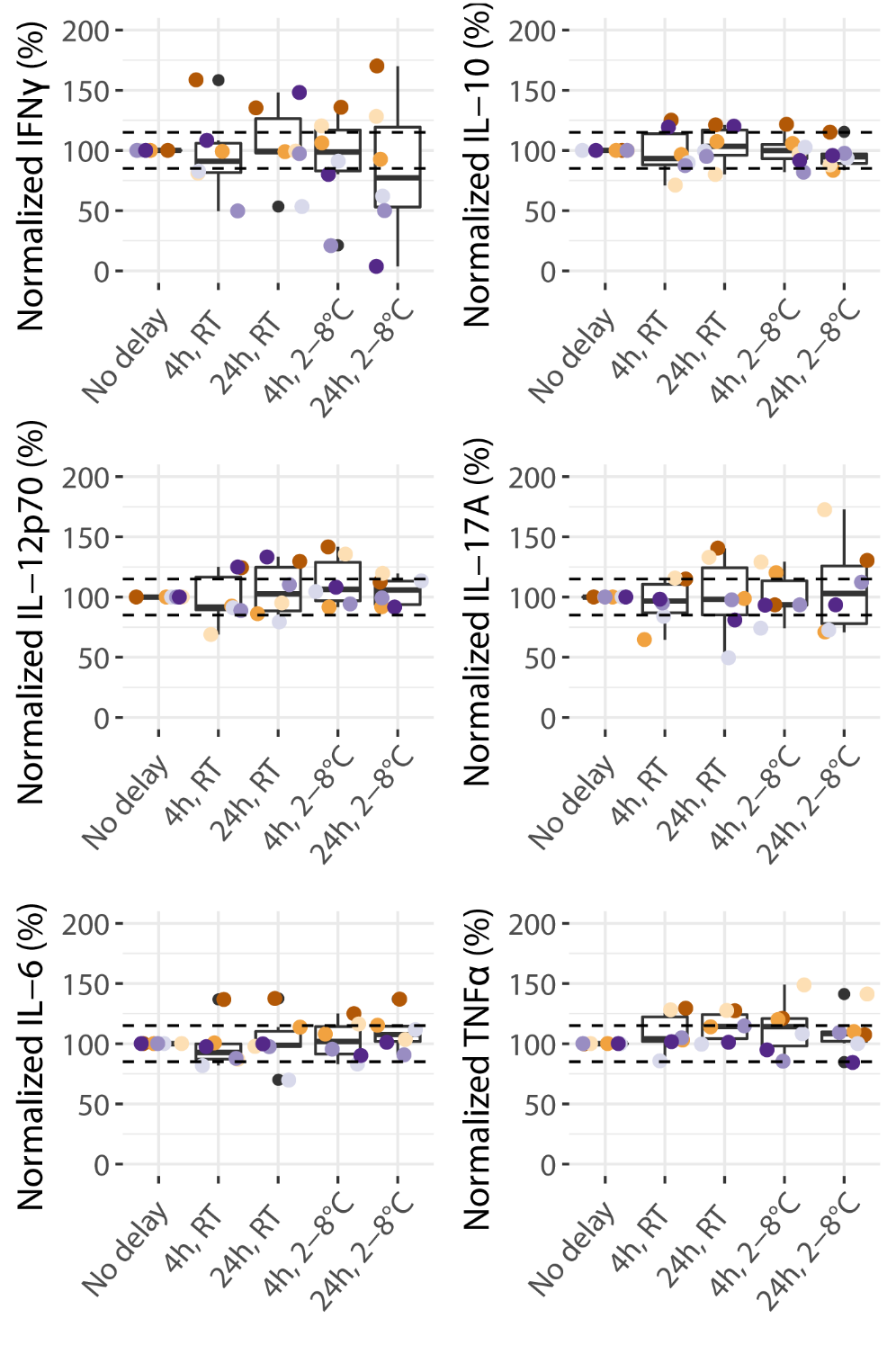
***

**Supplementary figure 3. Cytokine levels in EDTA plasma samples upon delayed storage post-centrifugation.***EDTA samples underwent delayed storage post-centrifugation and aliquoting (4h or 24h), while kept at RT or at 2-8°C. Levels were normalized against the levels measured in the samples that were stored immediately after centrifugation and aliquoting. Data points are color coded for subject (n=6). Horizontal reference lines are fixed at 85% and 115%. IFN=interferon, IL=interleukin, TNF=tumor necrosis factor, RT=room temperature.*


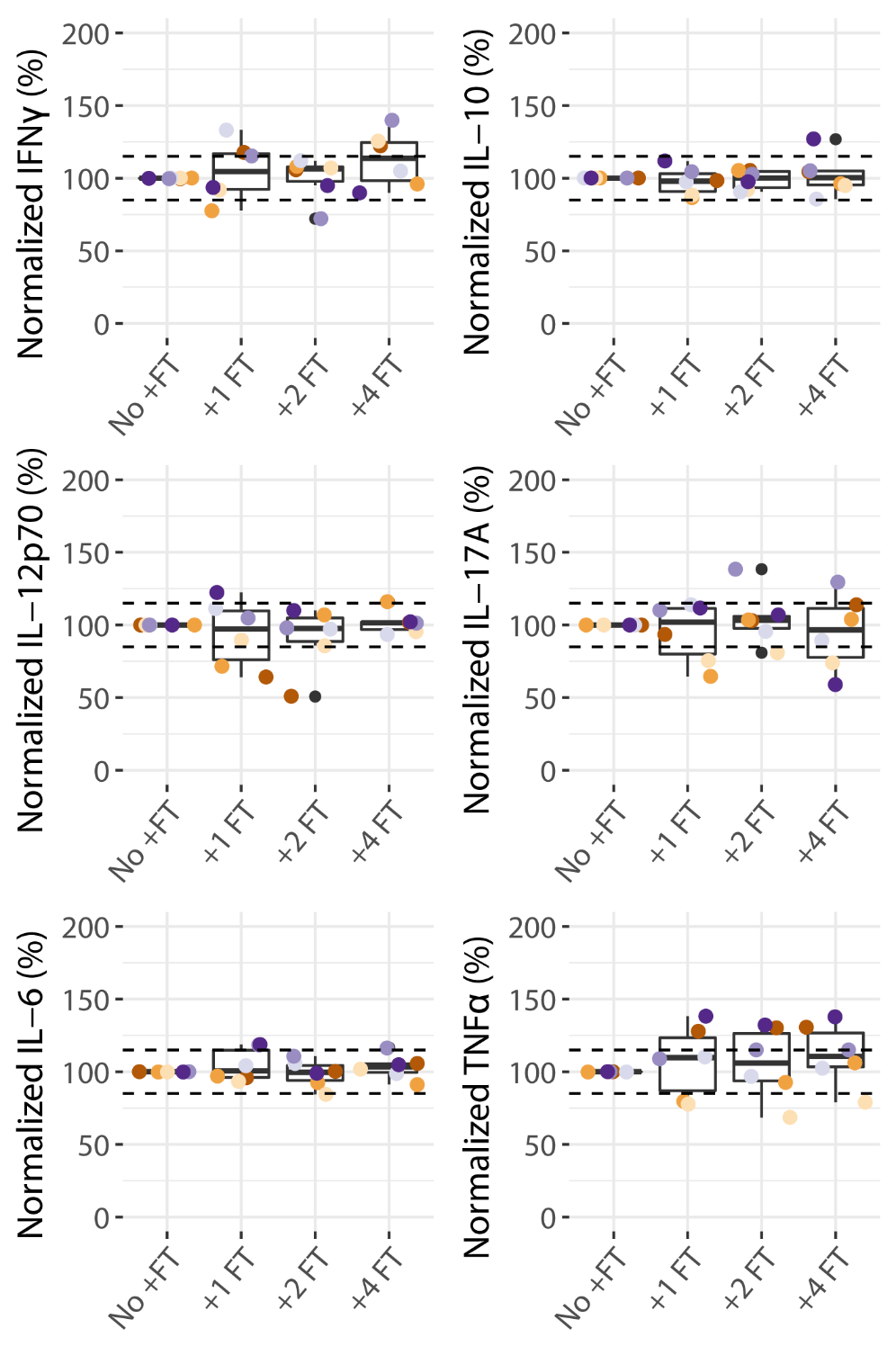


**Supplementary figure 4. Cytokine levels in EDTA plasma samples exposed to up to four freeze-thaw cycles.**
*EDTA samples were exposed to up to four additional freeze-thaw cycles. Levels were normalized against the levels measured in the samples that were not exposed to additional freeze-thaw cycles. Data points are color coded for subject (n=6). Horizontal reference lines are fixed at 85% and 115%. IFN=interferon, IL=interleukin, TNF=tumor necrosis factor, FT=freeze-thaw cycle.*
